# Sevoflurane Inhalation Accelerates the Long-Term Memory Consolidation via Small GTPase Overexpression in the Hippocampus of Mice in Adolescence

**DOI:** 10.1371/journal.pone.0163151

**Published:** 2016-09-15

**Authors:** Emi Nakamura, Hiroyuki Kinoshita, Guo-Gang Feng, Hisaki Hayashi, Maiko Satomoto, Motohiko Sato, Yoshihiro Fujiwara

**Affiliations:** 1 Department of Anesthesiology, Aichi Medical University School of Medicine, Nagakute, Aichi, Japan; 2 Department of Pharmacology, Aichi Medical University School of Medicine, Nagakute, Aichi, Japan; 3 Department of Physiology, Aichi Medical University School of Medicine, Nagakute, Aichi, Japan; 4 Department of Anesthesiology, Graduate School of Medical and Dental Sciences, Tokyo Medical and Dental University, Bunkyo-ku, Tokyo, Japan; Universita degli Studi dell'Insubria, ITALY

## Abstract

Sevoflurane exposure impairs the long-term memory in neonates. Whether the exposure to animals in adolescence affects the memory, however, has been unclear. A small hydrolase enzyme of guanosine triphosphate (GTPase) rac1 plays a role in the F-actin dynamics related to the synaptic plasticity, as well as superoxide production via reduced nicotinamide adenine dinucleotide phosphate (NADPH) oxidase activation. The current study was designed to examine whether sevoflurane exposure to mice in early adolescence modifies the long-term learning ability concomitantly with the changes in F-actin constitution as well as superoxide production in the hippocampus according to the levels of rac1 protein expression. Four-week-old mice were subjected to the evaluation of long-term learning ability for three days. On day one, each mouse was allowed to enter a dark chamber for five min to acclimatization. On day two, the procedure was repeated with the addition of an electric shock as soon as a mouse entered the dark chamber. All mice subsequently inhaled 2 L/min air with (Sevoflurane group) and without (Control group) 2.5% sevoflurane for three hours. On day three, each mouse was placed on the platform and retention time, which is the latency to enter the dark chamber, was examined. The brain removed after the behavior test, was used for analyses of immunofluorescence, Western immunoblotting and intracellular levels of superoxide. Sevoflurane exposure significantly prolonged retention time, indicating the enhanced long-term memory. Sevoflurane inhalation augmented F-actin constitution coexisting with the rac1 protein overexpression in the hippocampus whereas it did not alter the levels of superoxide. Sevoflurane exposure to 4-week-old mice accelerates the long-term memory concomitantly with the enhanced F-actin constitution coexisting with the small GTPase rac1 overexpression in the hippocampus. These results suggest that sevoflurane inhalation may amplify long-term memory consolidation via the increased cytoskeleton constitution in the hippocampus of animals in early adolescence.

## Introduction

The exposure of volatile anesthetics including sevoflurane or isoflurane to neonatal rodents at postnatal day 6 to 14 induces the long-term learning disabilities resulting from neurodegeneration [1–3]. The effects of these anesthetics in 7- to 16-week-old adult rodents are inconclusive, including impairment [4, 5], no change [3, 6] or augmentation of long-term memory [2, 7]. It has been, however, unclear whether the inhalation of a volatile anesthetic agent during adolescence, which is defined as 4- to 6-week-old in rodents [8], affects the long-term memory.

The hippocampus plays a critical role in the consolidation of memory [9, 10]. Modulation of F-actin dynamics induces cytoarchitecture changes associated with synaptic plasticity, resulting in the long-term memory formation as the actin is one of the main cytoskeletal proteins in the pre- and postsynaptic terminals [11, 12]. A small hydrolase enzyme of guanosine triphosphate (GTPase) rac1 is essential to evoke the long-term memory resulting from neurogenesis and dendritic spine formation via the enhanced F-actin constitution [12–14]. On the other hand, this small GTPase is known as a critical cytosolic subunit of reduced nicotinamide adenine dinucleotide phosphate (NADPH) oxidase, which produces superoxide upon the activation by recruitment toward the cellular membrane in many pathological conditions [15–17]. Our recent study has documented that the non-selective NADPH oxidase inhibition prevents the long-term memory impairment in mice neonatally exposed to sevoflurane, suggesting the idea that the rac1 protein overexpression may damp the cognitive function via increased oxidative stress in animals undergoing anesthesia [18].

Therefore, the current study was designed to examine whether sevoflurane inhalation to 4-week-old mice in the early adolescence accelerates the long-term learning ability concomitantly with changes in the enhanced F-actin cytoskeletal constitution coexisting with small GTPase rac1 overexpression in the hippocampus and whether the overexpression relates tissue levels of superoxide in this model.

## Materials and Methods

### Animals and ethical statement

The Animal Care and Use Committee of Aichi Medical University approved the protocol in the current study (2013–38). Four-week-old male C57BL/6 mice (SLC Japan Inc., Shizuoka, Japan) were used in this study and were housed in groups of five in a 12-h light-dark cycle (light from 7:00 to 19:00). Room temperature was maintained at 21 ± 1°C. All mice had ad libitum access to water and food. The current study employed only male mice to avoid potential variability caused by the estrous cycle [19].

### Long-term memory evaluation

Mice were subjected to a passive avoidance test (PAT), which can evaluate a conditional contextual response related to hippocampus-dependent memory [5, 20]. The test was carried out during the daytime between 9:00 and 12:00 for consecutive three days [20]. The apparatus consisted of an illuminated platform and a dark chamber separated by a guillotine door and it was equipped with Smart 3.0^™^ video tracking system (Panlab, S.U.L., Barcelona, Spain).

On day one, each mouse was placed on an illuminated platform and allowed to enter a dark chamber for five minutes to acclimatization [20]. On day two, the procedure was repeated with the addition of an electric shock (0.6 mA) for seven seconds as soon as a mouse entered the dark chamber [20]. All mice subsequently inhaled 2 L/min air with (Sevoflurane group) and without (Control group) 2.5% sevoflurane for three hours in the incubator heated to 38°C [20]. The concentration of sevoflurane was selected to obtain sedative, but not surgical, anesthetic condition considering the minimum alveolar anesthetic concentration of sevoflurane that produces immobility in 50% of subjects given a noxious stimulation in mice has been reported as about 3.4% [21]. On day three, each mouse was again placed on the platform [20]. The behavior of each mouse was recorded and evaluated using the video tracking system. Acquisition or retention time (sec) defined the latency to enter the dark chamber on day two and the latency to enter it on day three, respectively [20]. The locomotor activity as the velocity of movement (cm/sec) in the illuminated platform was also evaluated [22].

### Hippocampus isolation

Mice immediately after all of the behavior tests were euthanatized by the cervical dislocation. The CA1 region of the hippocampus, which plays a central role in the long-term memory consolidation [23, 24], was isolated using the mouse brain atlas [25] and was used for analyses of Western immunoblotting, immunofluorescence and in situ superoxide production. The coronal sections of the whole mice brain from -2 to 3 mm from the Bregma were used for immunofluorescence and in situ superoxide production.

### Immunohistochemical analysis

The isolated brain was immersed in 4% paraformaldehyde, immersed in phosphate-buffered saline (pH 7.4) overnight at 4°C [26]. Twenty-μm-thick coronal sections of the brain were cut on a cryostat, mounted onto microscope slides and dried at 37°C for three hours. The fixed sections were subsequently exposed to phosphate-buffered saline with 3% bovine serum albumin in combination with 0.05% Triton X-100 at 24°C for 60 min [26]. For immunohistochemical determination of target molecules, the sections were incubated for 30 min in the dark with the Alexa Fluor^®^ 488 Phalloidin (5 U/mL; Thermo Fisher Scientific Corp., Carlsbad, CA, USA) [27]. Finally, 4,6-dianidina-2-phenylindole (DAPI, one μg/mL; Thermo Fisher Scientific Corp., Carlsbad, CA, USA) was applied for five min to stain all nuclei [27]. Images of cellular fluorescence were acquired using a microscope fitted with BZ-II analyzer software (Model BZ-9000 Generation II, Keyence, Osaka, Japan). Settings were adjusted based on the fluorescence intensity in tissues from the Control group and were identical for the acquisition of images from all of the tissues. The negative control did not show any nonspecific staining. The total F-actin green fluorescence was determined by subtracting that of background in each specimen. Six fields of view were analyzed using three sections from different animals (two fields from each) of the hippocampus CA1 region.

### Western immunoblotting analysis

The isolated hippocampus was quickly frozen at -80°C. Cytosolic and membranous fractions were prepared and used for Western immunoblotting analysis [28]. The frozen hippocampus was homogenized by the Polytron homogenizer in 300 μl cell permeabilization buffer (10 mmol/L Tris [pH7.4], 1 mmol/L ethylenediaminetetraacetic acid (EDTA), 200 mmol/L sucrose with protease inhibitor) [26]. The lysate was centrifuged at 900 × g for 10 min at 4°C to remove debris [26]. The supernatant, which was centrifuged again at 100,000 × g for 60 min at 4°C, was used as the cytosolic fraction [26]. The pellet, which was dissolved in 150 μl of solubilization buffer (10 mmol/L Tris [pH7.4], 1 mmol/L EDTA, 0.5% Triton-X with protease inhibitor), was used as the membrane fraction [26]. Proteins were separated by sodium dodecyl sulfate-poly-acrylamide gel electrophoresis and transferred to polyvinylidene difluoride membranes (Immobilon^™^-Transfer Membrane, Millipore Corp., Billerica, MA, USA) [26]. These membranes were assessed with antibodies against rac1 (cat. #33186, 1:2000 dilution; Abcam plc, Cambridge, UK), α-Tubulin (cat. #80779, 1:2000 dilution; Abcam plc, Cambridge, UK) and sodium-potassium adenosine triphosphatase (Na^+^/K^+^ ATPase, cat. # 3010, 1:2000 dilution; Cell Signaling Technology Inc., Danvers MA). The results were quantified based on the expression level of Na^+^/K^+^-ATPase or α-Tubulin using the Image J software.

### Measurements of in situ superoxide production

An oxidative fluorescent dye hydroethidine (Polyscience Inc., Warrington PA, USA) was used for semi-quantitative evaluation of superoxide in situ [15]. Hydroethidine (7 mg) was diluted with N,N-dimethylacetamide (1 mL). The whole brain was quickly frozen at −80°C. Twenty-μm-thick coronal sections of the brain were cut on a cryostat and mounted onto microscope slides [18]. Each slice was incubated with hydroethidine (2×10^−6^ mol/L) in a light-protected chamber at 37°C for 20 min [15]. Hoechst 33258 (1 μg/mL, Nacalai Tesque, Kyoto, Japan) was simultaneously applied to stain nuclei of cells. Images of cellular fluorescence were acquired using a microscope fitted with BZ-II analyzer software (Model BZ-9000 Generation II, Keyence, Osaka, Japan). Settings were adjusted based on the fluorescence intensity in tissues from the Control group and were identical for the acquisition of images from all of the tissues. The negative control did not show any nonspecific staining. The total ethidium bromide fluorescence was determined by subtracting that of background in each specimen. Six fields of view were analyzed using three sections from different animals (two fields from each) of the hippocampus CA1 region.

### Statistical Analysis

The power calculation was done using Sample Power 3.0^™^ (IBM Japan Inc., Tokyo, Japan). In the current study, a sample size of 16 gave 86% power to the detected change in time to the dark chamber of 76 sec at a significance level of 0.05 (SD = 69). Statistical analysis was performed using PASW Statistics 18^™^ (IBM Japan Inc., Tokyo, Japan). The data were expressed as the means ± SD and were analyzed by one-way analysis of variance (ANOVA) with Scheffe’s test. Differences were considered to be statistically significant when P is < 0.05.

## Results

### Long-term memory evaluation

**Fig 1:**
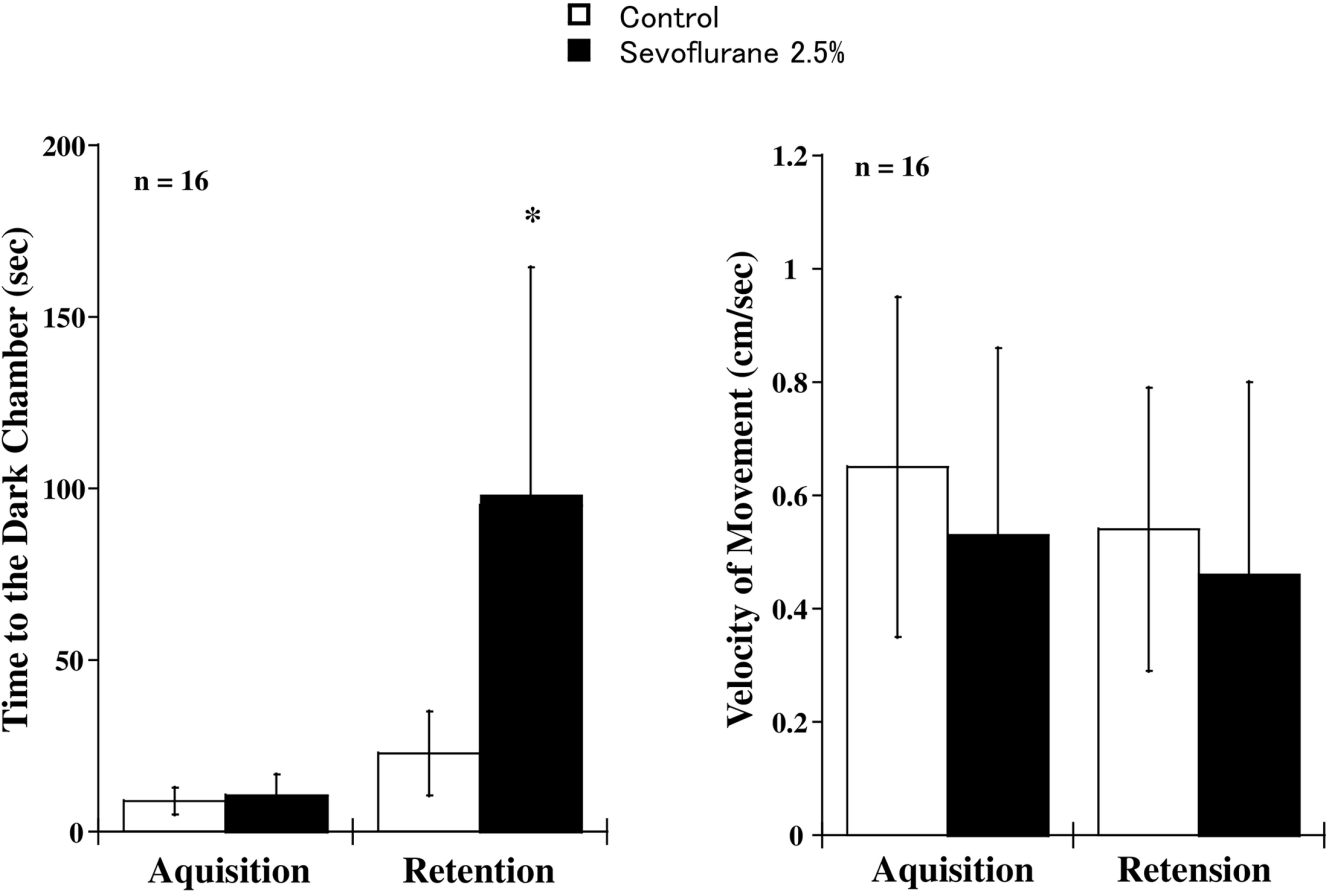
The effect of 2.5% sevoflurane inhalation on the long-term memory formation. Mice in the Sevoflurane (n = 16) and Control (n = 16) groups were subjected to PAT, which can evaluate a conditional contextual response related to hippocampus-dependent memory. Acquisition or retention time (sec) defined the latency to enter the dark chamber on day two and the latency to enter it on day three, respectively. The velocity of movement (cm/sec) in the illuminated platform on day two and day three was also evaluated to rule out the effect of sevoflurane on the locomotor activity. The data were expressed as the means ± SD and were analyzed by one-way ANOVA with Scheffe’s test. *: Differences between Sevoflurane and Control groups are statistically significant (P<0.001). The acquisition time (P = 0.319), the velocity of movement on day two (P = 0.281) and day three (P = 0.418) did not differ between the Sevoflurane and Control groups.

Sevoflurane exposure (2.5% for three hours) significantly prolonged retention time, which is the latency to enter the dark chamber on day three, whereas the acquisition time, that is the latency to enter it on day two did not differ between the Sevoflurane and Control groups (Fig 1). There was, however, no difference in the locomotor activity shown as the velocity of movement in the illuminated platform on day two and day three between the Sevoflurane and Control groups (Fig 1).

### Immunohistochemical analysis

**Fig 2:**
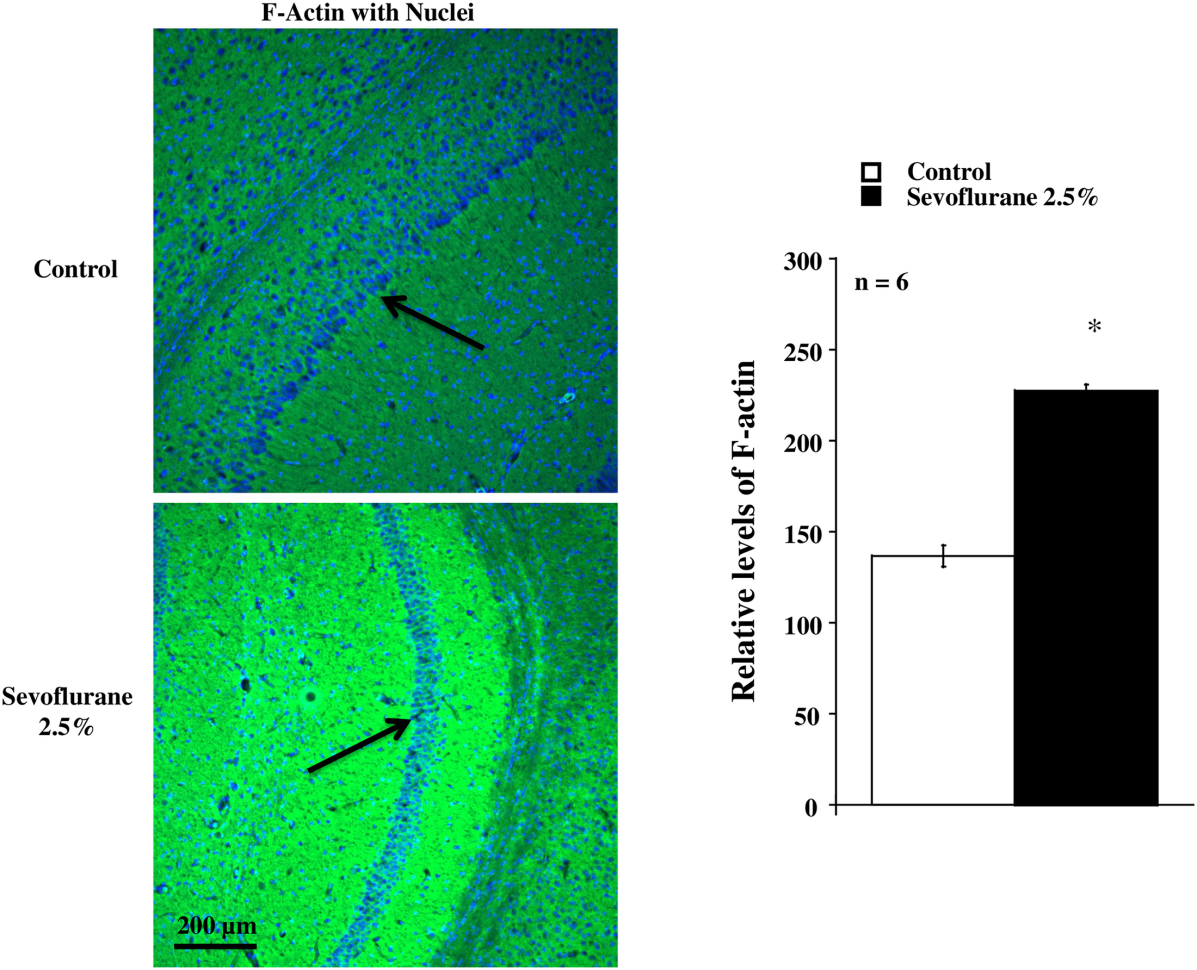
Immunofluorescent image analysis in the hippocampus from mice with (Sevoflurane) or without (Control) sevoflurane inhalation. **(Left)** The representative images in Sevoflurane and Control groups are shown. Note augmented the green fluorescence in the stratum radiatum and stratum oriens of the hippocampus CA1 region indicating the F-actin constitution in the Sevoflurane group. The black arrows indicate nuclei of pyramidal cells. **(Right)** The cumulative data of immunofluorescent image analysis is shown. The data were expressed as the means ± SD and were analyzed by one-way ANOVA. *Differences between Sevoflurane and Control groups are statistically significant (P<0.001).

Sevoflurane inhalation 2.5% for three hours augmented the F-actin constitution in the stratum radiatum and stratum oriens of the hippocampus CA1 region after completion of the behavior tests (Fig 2).

### Western immunoblotting analysis

**Fig 3:**
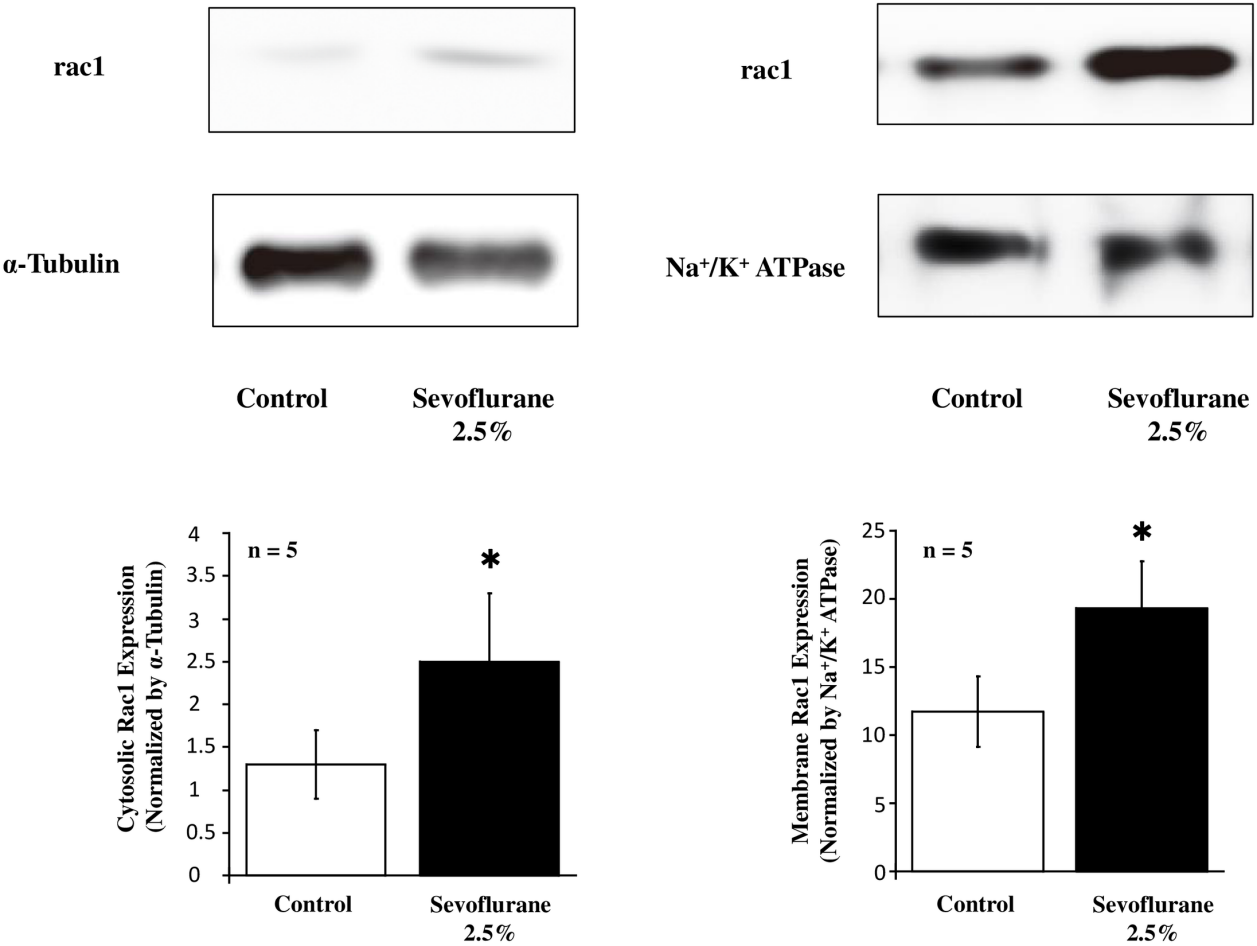
The Western immunoblotting analysis in the hippocampus from mice with (Sevoflurane) or without (Control) sevoflurane inhalation. In the bar graph, the band density in the cytosolic (**Left**) and membrane (**Right**) fractions was expressed as means ± SD and the data were analyzed by one-way ANOVA. The results were quantified based on the expression level of Na^+^/K^+^-ATPase or GAPDH. *Differences between Sevoflurane and Control groups are statistically significant (P = 0.15 and P = 0.005 for cytosolic and membrane rac1 protein expression, respectively).

Sevoflurane inhalation significantly enhanced the protein overexpression of a small GTPase rac1 in the cytosolic, as well as the membrane, fraction of the hippocampus after completion of the behavior tests (Fig 3).

### Measurements of in situ superoxide production

**Fig 4:**
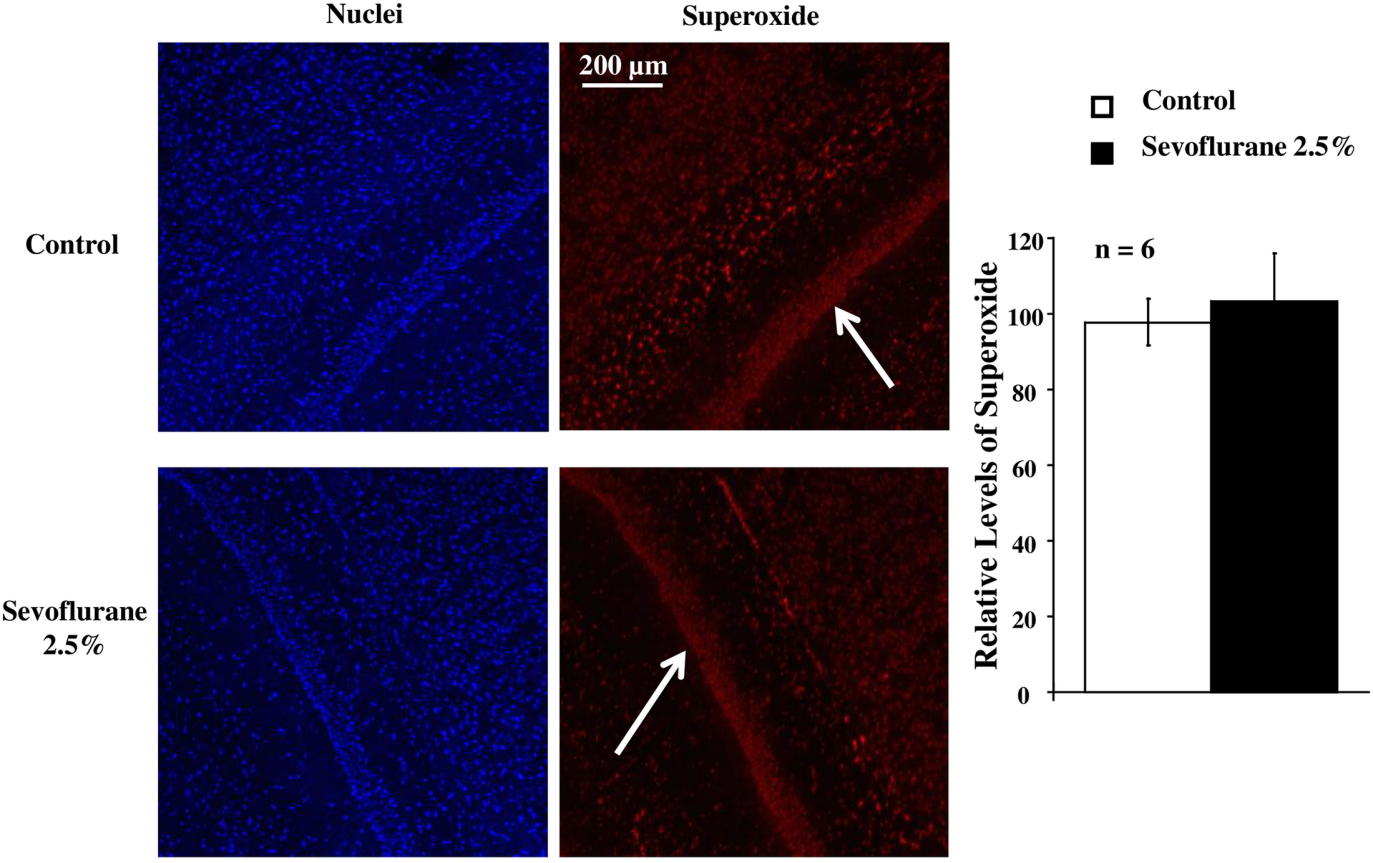
Measurements of in situ superoxide production in the hippocampus from mice with (Sevoflurane) or without (Control) sevoflurane inhalation. **(Left)** Representative images of in situ superoxide production in the hippocampus of mice with or without sevoflurane inhalation are shown. Blue or red fluorescence indicates nuclei and superoxide in the neuronal cells, respectively. The white arrows indicate nuclei of pyramidal cells in the hippocampus CA1 region. (**Right**) In the bar graph, levels of relative superoxide production in the hippocampal pyramidal cells were expressed as means ± SD and the data were analyzed by one-way ANOVA. The relative levels of superoxide did not differ between the Sevoflurane and Control groups (P = 0.365).

The levels of superoxide in pyramidal cells of the hippocampus CA1 region after completion of the behavior tests did not differ between the Sevoflurane and Control groups (Fig 4).

## Discussion

The postnatal day from 30 to 45 in rodents best defines adolescence judged from the age-dependent behavior including increased locomotor and explorative activities [8]. The range corresponds to that around 10 to less than 20 yrs in humans where the brain achieves developmental milestones of the maximum volume of gray matter as well as cortical thickness [8]. In the current study, 2.5% sevoflurane exposure, which is a sedative dose in the rodents, unexpectedly prolonged retention time in the mice at the postnatal day 30, indicating the role of sevoflurane inhalation in the augmented conditional contextual response related to hippocampus-dependent memory [5, 9, 10, 20]. These results are clearly different from those previous studies on neonatal rodents at postnatal day 6 to 14, in which the exposure of volatile anesthetics including sevoflurane or isoflurane impaired the long-term learning abilities of these animals [1–3]. The effects of these anesthetics on the long-term memory in the young adult animals, which are 7- to 16-week-old, are variable and inconsistent [2–7]. At least, it can be concluded that adolescence is a time window in rodents when the volatile anesthetic inhalation enhances the long-term memory although the mechanisms about such the differential effect on the memory formation among time periods in the lifetime remain unanswered. In the current study, the locomotor activity of day two and day three shown as the velocity of movement in the illuminated platform did not differ between the Sevoflurane and Control groups. These results rule out the possibility that sevoflurane inhalation modifies the locomotor activity resulting in prolongation of the time to the dark chamber at the retention phase.

In the present study, the sevoflurane inhalation augmented the F-actin constitution in the hippocampus where contributes to the memory consolidation [9, 10]. The F-actin, which is one of the major cytoskeletal proteins in the pre- and postsynaptic terminals, plays a critical role in the dendritic spines formation related to the synaptic plasticity necessary to the long-term memory [11, 12]. Indeed, previous studies demonstrated that the intrahippocampal infusion of the F-actin cytoskeleton assembly inhibitors impairs the consolidation of contextual fear memory, indicating the importance of actin rearrangements in the memory formation [29]. These results support the conclusion that sevoflurane exposure in early adolescence enhances the long-term memory formation.

Small GTPases are proteins, which regulate the actin cytoskeleton in the central nervous system, play roles in brain development and function, as well as cognitive function [12, 13]. Of these, Rac1 is essential to evoke the long-term memory resulting from neurogenesis and dendritic spine formation via the enhanced F-actin constitution [12–14]. Sevoflurane inhalation significantly enhanced the protein overexpression of rac1 in the cytosolic, as well as the membrane, fraction of the hippocampus after completion of the behavior tests in the current study. These results suggest that the small GTPase contributes to the long-term memory formation induced by sevoflurane inhalation via the increased F-actin constitution in the hippocampus. Further studies targeting the relation between spine morphology of the pyramidal neurons in the CA1 region of the hippocampus and the intracellular rac1 protein expression require proving this conclusion [24].

Importantly, rac1 is also the critical cytosolic subunit of NADPH oxidase, which produces superoxide in many pathological conditions [15–17]. The non-selective NADPH oxidase inhibition achieved by apocynin prevents the long-term memory impairment in mice neonatally exposed to sevoflurane, suggesting that the rac1 protein overexpression may rather damp the cognitive function via increased oxidative stress in animals undergoing anesthesia [18]. In the current study, the levels of superoxide in the hippocampus did not differ between the Sevoflurane and Control groups. These results indicate that the rac1 protein overexpression induced by sevoflurane in rodents at the early adolescence does not cause oxidative stress in the brain although the reason for the differential role of the small GTPase in the time windows between neonates and early adolescence has been unclear. This conclusion is consistent with our previous study that volatile anesthetics are most likely inhibitors to another cytosolic subunit of NADPH oxidase, p47phox [30]. It is, however, crucial to note that the NADPH oxidase activity is essential to support hippocampus-dependent learning and memory, as well as normal performance in behavioral paradigms that require other brain regions [31]. Therefore, the modulation of superoxide so as not to produce oxidative stress appears necessary to maintain normal brain function.

Whether the sevoflurane inhalation during adolescence affects the long-term memory in humans as well as rodents is unclear. A recent clinical study has demonstrated that general anesthesia using propofol, but not sevoflurane, impairs the memory lasting a week postoperatively in children aged between 7 and 13 years [32]. The study results indicate the potential significance of current findings that sevoflurane inhalation may play a role in the memory consolidation especially in adolescence. Further clinical studies are certainly needed to clarify the effect as well as the mechanisms of anesthetics on cognition in this population.

This study is the first to demonstrate that sevoflurane exposure to 4-week-old mice accelerates the long-term memory concomitantly with the enhanced F-actin constitution coexisting with the small GTPase rac1 overexpression in the hippocampus. These results suggest that sevoflurane inhalation in early adolescence amplifies long-term memory consolidation via the increased cytoskeleton constitution in the hippocampus although the clinical relevance is still unclear.

## Supporting Information

S1 Fig Whole Western Blots in the hippocampus from mice with (Sevoflurane) or without (Control) sevoflurane inhalation. Please refer to protein size markers in the figure.
(PPTX)

## Abbreviations

ANOVA: analysis of variance
GTPase: small hydrolase enzyme of guanosine triphosphate
NADPH: reduced nicotinamide adenine dinucleotide phosphate
PAT: passive avoidance test
