# Supplementary figures and images for: Sevoflurane Inhalation Accelerates the Long-Term Memory Consolidation via Small GTPase Overexpression in the Hippocampus of Mice in Adolescence
Source: PLoS One. 2016 Sep 15;11(9):e0163151. doi: 10.1371/journal.pone.0163151 (PMC5025001; doi:10.1371/journal.pone.0163151)

## Slide 1
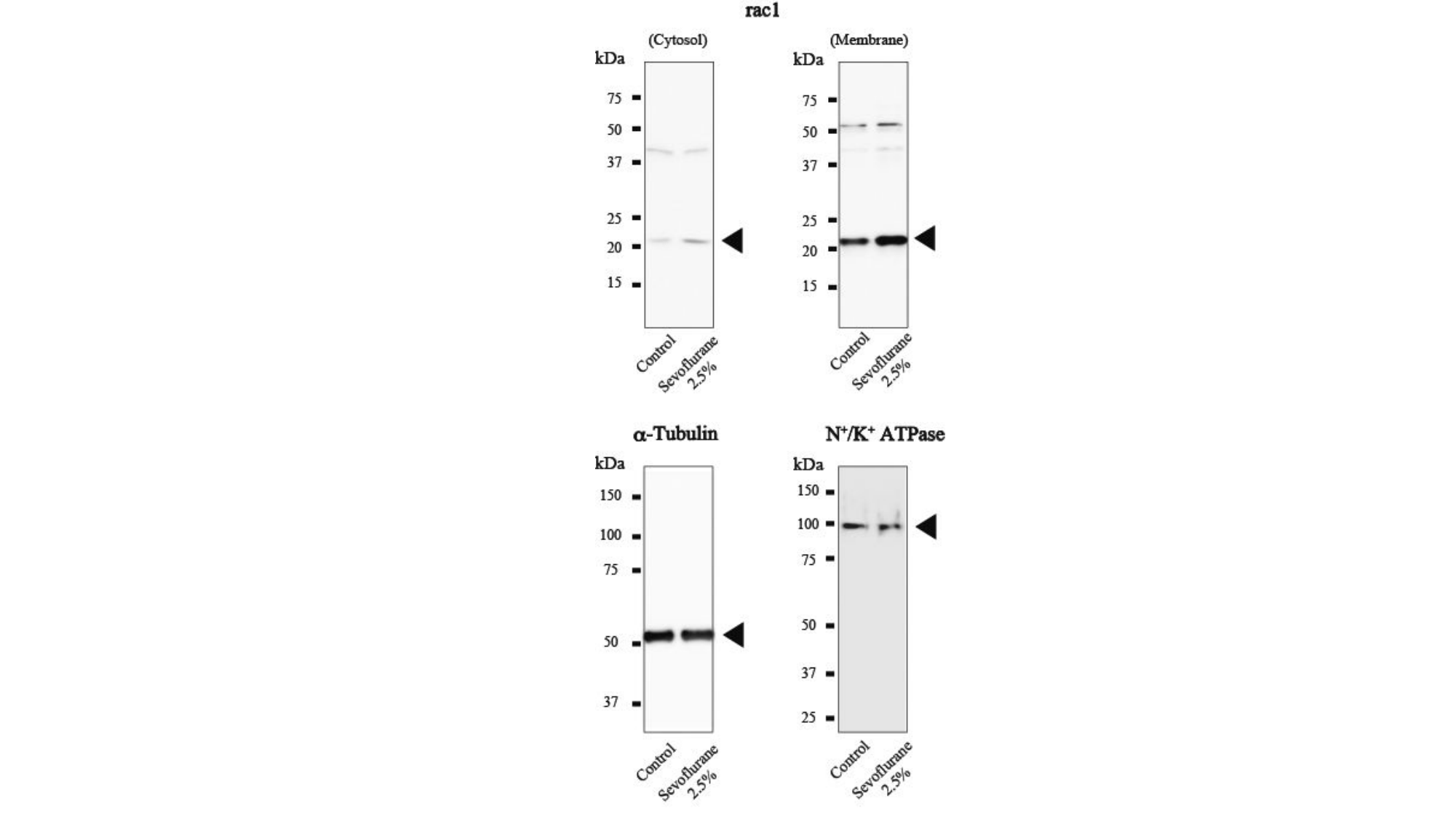

Supplement: S1 Fig — Please refer to protein size markers in the figure. (PPTX) [file pone.0163151.s001.pptx]
